# Recent advances in understanding and managing hairy cell leukemia

**DOI:** 10.12688/f1000research.13265.1

**Published:** 2018-04-27

**Authors:** Tobias Roider, Brunangelo Falini, Sascha Dietrich

**Affiliations:** 1 Department of Medicine V, University of Heidelberg, Heidelberg, Germany; 2 Institute of Hematology and Center for Hemato-Oncology Research (CREO), University and Hospital of Perugia, Perugia, Italy

**Keywords:** hairy cell leukemia, BRAF, vemurafenib

## Abstract

Hairy cell leukemia is a rare B-cell malignancy that is characterized by an indolent course. It was initially described as a distinct entity in 1958. Before the establishment of modern treatment, median survival was only 4 years. Since then, major advances in the treatment and understanding of the biology and genomic landscape of hairy cell leukemia have been made. This review summarizes the present understanding of hairy cell leukemia with particular focus on the development of novel and targeted approaches to treatment.

## Introduction

Hairy cell leukemia (HCL) is a rare mature B-cell malignancy, which was initially described by Bouroncle *et al*. in 1958 ^1^. HCL has an incidence of 0.3 cases per 100,000 individuals ^2^. It occurs about four times more often in men than in women, with a median age at diagnosis of 55 years ^3^. HCL cells are characterized by thin cytoplasmic hair-like projections, giving the disease its name ^4^. Leukemic hairy cells accumulate in the bone marrow and cause pancytopenia, which is the most common finding at initial presentation. HCL patients report symptoms of fatigue, infections, and, occasionally, left-sided abdominal pain caused by splenomegaly. In contrast to many B-cell malignancies, lymphadenopathy is rare in HCL patients ^5^. A severe fibrotic reaction is commonly found in the bone marrow of HCL patients, which often complicates a diagnostic bone marrow aspiration. The diagnosis is usually based on the detection of typical morphological features and a unique immunophenotype with flow cytometry of peripheral blood and/or histological and immunohistochemical (IHC) analysis of trephine biopsies. HCL cells show a characteristic gene expression profile signature that points to their origin from memory B cells ^6, 7^. Although standard treatment with purine analogues is very effective in the majority of patients with HCL, there is a small subset of relapsed and refractory HCL patients who qualify for investigational therapies with monoclonal antibodies or small molecule compounds ^8^. In this review, we will discuss these novel therapeutic agents as well as recent advances in understanding the molecular pathogenesis of HCL.

## The biology of HCL

In 2011, Tiacci *et al*. discovered that classical HCL is characterized by a gain-of-function mutation of the BRAF serine/threonine protein kinase (V600E) ^9, 10^. In the initial validation series, all HCL patients showed this particular mutation, while a set of 195 B-cell lymphomas and leukemias did not harbor a mutated *BRAF* gene. The vast majority of *BRAF*-V600E mutations in HCL are heterozygous. Homozygous mutations are rare but have been suggested to be associated with a more aggressive disease course ^11^. Recurrent deletions of the *BRAF* gene locus on chromosome 7q34 have been described in HCL and lead to loss of heterozygosity ^12^. *BRAF* mutations, different from V600E, seem to be extremely rare in HCL and have been described in only two patients so far ^13^. The incidence of *BRAF* mutations in nearly 100% of HCL cases at diagnosis (encompassing the whole disease spectrum), their somatic nature, their presence in the entire tumor clone, and their high stability at relapse strongly suggest that the pathogenesis of HCL critically depends on constitutively activated BRAF ^10, 14, 15^.

Chung *et al*. reported that *BRAF*-V600E mutations are already present in hematopoietic stem cells (HSCs) or B-cell lymphoid progenitors of HCL patients and that these patients exhibit marked alterations in hematopoietic stem/progenitor cell (HSPC) frequencies ^16^. Transplantation of *BRAF*-V600E-mutant HSCs from an HCL patient into immunodeficient mice resulted in stable engraftment of *BRAF*-V600E-mutant human hematopoietic cells, revealing the functional self-renewal capacity of HCL HSCs. However, none of the transplanted mice developed typical HCL, strongly suggesting that the development of a full HCL phenotype may require a permissive epigenetic background (likely restricted to a particular stage of B-cell differentiation) and/or the acquirement of further genetic lesions ^16^.

**Figure 1.**
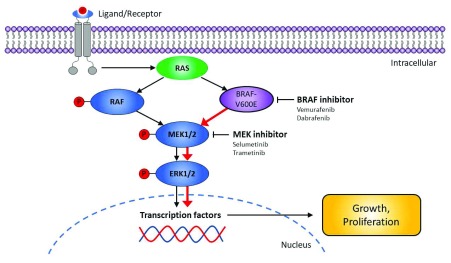
RAF-MEK-ERK signaling pathway in hairy cell leukemia. The figure shows the RAF-MEK-ERK signaling pathway in hairy cell leukemia and highlights targets for therapeutic intervention.

The *BRAF*-V600E mutation constitutively activates BRAF, providing oncogenic signaling through the MEK-ERK cascade ^10, 17^ ( Figure 1). Both *in vitro* and *in vivo* studies have demonstrated that BRAF-dependent phospho-ERK activation is a critical signaling event in HCL ^10, 18, 19^. Moreover, *in vitro* treatment of primary purified HCL cells with BRAF and MEK inhibitors has resulted in marked dephosphorylation of MEK/ERK, silencing of the RAF-MEK-ERK pathway transcriptional output, loss of the specific HCL gene expression profile signature, change of the characteristic morphology of the leukemic cells (from “hairy” to “smooth”), and eventually apoptosis ^14, 15, 20^.

Aberrant expression of cell cycle-related proteins such as cyclin D1 has been shown to be reversible using inhibitors of activated BRAF signaling, suggesting that expression is not a constitutive disease trait but elicited by MEK/ERK signaling and oncogenic BRAF mutations, respectively ^18^. This could have a significant effect on the assessment of minimal residual disease (MRD) when considering inhibitor treatment because the profile of the marker cyclin D1 might be dynamic, as well as on targeted drug therapy, which may be shortened due to the on-target effect of inhibitors.

## Differential diagnosis of HCL

Historically, there were two different forms of HCL: the more-common classical HCL (90%) and the less-frequent HCL variant (10%). HCL variant is characterized by a more aggressive disease course and poor response to purine analogs ^21^. Most importantly, HCL variant cases are commonly negative for *BRAF*-V600E mutation, indicating that HCL variant is a biologically distinctive entity. A small subset of patients with bona fide classical who also do not harbor any *BRAF* mutation has been reported only in a single study ^22^. However, these cases are often characterized by an IGHV4-34 immunoglobulin rearrangement, which is in general absent in classic HCL and is associated with as poor a prognosis as HCL variant ^22^.

Almost 50% of HCL-variant and IGHV4-34-expressing HCL cases were found to harbor activating mutations in the *MAP2K1* gene encoding MEK1 ^23^. All but one of the identified mutations (n=15) have been described and are known to strongly increase phospho-ERK levels and consequently cell proliferation ^23^. These findings underline the importance of constitutive MEK-ERK signaling, even in this HCL-like disorder.

HCL cells typically show a distinctive immunophenotype co-expressing CD19, CD20, CD11c, CD25, CD103, and CD123. In contrast, HCL variant lacks the expression of CD25 and CD123 ^24^. Moreover, HCL cells strongly express CD200, which can also be used as another distinctive marker to differentiate HCL ^25, 26^. *BRAF*-V600E is now regarded as a specific oncogenic mutation occurring only in HCL ^27^. Another distinctive feature of HCL is the expression of annexin A1, which is easily accessible by immunohistochemical staining ^28^. In addition to HCL variant, the 2016 revision of WHO classification of lymphoid neoplasms recognizes two other entities resembling HCL: splenic marginal zone lymphoma (SMZL), usually associated with *NOTCH2* mutations, and splenic diffuse red pulp small B-cell lymphoma (SDRPBCL), still listed as a provisional entity, whose genomic landscape has not been yet clarified ^27^. Table 1 summarizes the most important differential diagnoses of HCL and their characteristic markers.

**Table 1.** Differential diagnoses in HCL and their characteristic features.

	HCL	HCL variant	SMZL	SDRPBCL
**Frequency**	0.3/100,000	0.03/100,000	0.13/100,000	n/a
**Ratio m:f**	4:1 (m:w)	1–2:1 (m:w)	1:3 (m:w)	1–2:1 (m:w)
**Median age**	50–55	>70	65–70	65–75
**Lymphocytosis**	≤10%	≥90%	≥50%	≥50%
**Immunophenotype**	CD11c ^+^ CD103 ^+^ CD25 ^+^ CD200 ^+^ CD23 ^-^ CD5 ^-^	CD11c ^+^ CD103 ^+/ ^-^^ CD25 ^-^ CD200 ^-^ CD23 ^-^ CD5 ^-^	CD11c ^-^ CD103 ^+/ ^-^^ CD5 ^+/ ^-^^ CD200 ^+^ CD23 ^+/ ^-^^ CD5 ^+/ ^-^^	CD11c ^+^ CD103 ^-^ CD25 ^+/ ^-^^ CD23 ^-^ CD5 ^+/ ^-^^
**Immunohistochemistry**	DBA.44 ^+^ Cyclin D1 ^+^ Annexin A1 ^+^	DBA.44 ^+^ Cyclin D1 ^+/ ^-^^ Annexin A1 ^-^	DBA.44 ^+^ Cyclin D1 ^-^ Annexin A1 ^-^	DBA.44 ^+^ Cyclin D1 ^-^ Annexin A1 ^-^
**Genotype**	*BRAF*-V600E mutation	*BRAF* wildtype, ≈50% *MEK1* mutations, ≈50% IGHV4-34 rearrangement	*BRAF* wildtype, frequent *NOTCH2* mutations	*BRAF* wildtype

HCL, hairy cell leukemia; IGHV, immunoglobulin heavy-chain variable; SDRPBCL, splenic diffuse red pulp small B-cell lymphoma; SMZL, splenic marginal zone lymphoma

Testing for the *BRAF*-V600E mutation in routine clinical practice can be helpful as an additional marker if there is any diagnostic uncertainty. For relapsed and refractory patients, we strongly recommend evaluating the *BRAF* mutation status, since this may serve as a therapeutic target. The limited number of HCL cells present in the peripheral blood requires highly sensitive molecular assays to detect *BRAF* mutations (e.g. allele-specific polymerase chain reaction) ^9^. Alternatively, *BRAF*-V600E mutation-specific antibodies can be used for immunohistochemical staining in bone marrow biopsies ^29, 30^. However, further validation of the diagnostic utility of these reagents in a larger number of cases is required.

## Cooperating mutations of *BRAF*-V600E in HCL

In addition to the *BRAF*-V600E mutation, the most common genetic alteration in classical HCL was a copy number loss of chromosome 7q. The minimally deleted region of this copy number alteration includes the wild-type locus of *BRAF*. This genetic lesion subdivides individuals with classical HCL into those with hemizygous versus heterozygous mutations of *BRAF* ^12^. A whole-exome sequencing study of relapsed and refractory HCL patients revealed known cancer-associated genes, such as *EZH2* and *ARID1A,* as well as novel inactivating mutations of the cell cycle inhibitor *CDKN1B* (p27) ^31^. In a cohort of 81 mostly untreated HCL patients, the incidence of *CDKN1B* mutations was 16% ^31^. While a clinical impact of *CDKN1B* mutations was not found, the data identify *CDKN1B* as the second most commonly mutated gene in HCL. *CDKN1B* is a critical element of cell-cycle control and a known tumor suppressor in different solid cancers ^32^. *CDKN1B* prevents the activation of cyclin E-CDK2 or cyclin D-CDK4 complexes and thereby regulates cell-cycle progression in the G1 phase. Interestingly, BRAF-induced senescence in premalignant naevi is circumvented by deletion or mutation of *CDKN2A* in invasive melanoma ^33^. In *BRAF*-mutated hairy cell leukemia, *CDKN1B* loss may serve as a mechanism to escape oncogene-induced senescence ^31^. In addition to *CDKN1B* mutations cooperating with *BRAF*-V600E, recurrent, inactivating mutations in *KMT2C* ( *MLL3*) were identified in 15% and 13% of classical HCL and HCL variant, respectively ^12^. Another study described somatic mutations or deletions of the *Krüppel-like factor 2* ( *KLF2*) in 4 of 24 (16%) HCL patients examined ^34^, but *KLF2* mutations are more frequent in other B-cell malignancies, such as SMZL (31%) and diffuse large B-cell lymphoma (26%) ^34, 35^. Although we have better described the genetic landscape of HCL during recent years, the function of mutations cooperating with *BRAF*-V600E remains to be elucidated.

## Conventional therapeutic strategies in HCL

At initial diagnosis, most patients will require treatment owing to hematopoietic insufficiency. Accepted indications to start treatment are hemoglobin <11 g/dL, platelet count <100,000/µL, or absolute neutrophil count <1,000/µL. Less frequently, increased susceptibility to infections or symptomatic splenomegaly may also serve as criteria to start treatment ^5^. The introduction of the purine analogs cladribine and pentostatin into the treatment landscape of HCL significantly improved the outcome of HCL patients ^36^. Prospective randomized studies comparing pentostatin and cladribine as first-line treatment have not been conducted, but retrospective studies suggested equivalent activity of both drugs with induction of complete remission (CR) in approximately 85% of untreated patients ^37– 39^. The median treatment-free survival for patients with CR after treatment with purine analogs was more than 10 years in most studies ^40– 42^. In contrast, patients with a partial remission (PR) had a significantly shorter treatment-free survival of 3 years ^43, 44^. Although achievement of CR, beyond first-line treatment, is associated with similar good outcome, the proportion of patients with insufficient response and early relapse increases with each treatment round ^38, 44^. Apart from pentostatin and cladribine, there are hardly any effective, approved treatment options: interferon alpha, splenectomy, and rituximab monotherapy should be considered only in a small subset of patients ^5^. Recently, multiple biology-based treatment options have become available for refractory HCL patients, which will be discussed below.

## Therapeutic targeting of *BRAF*-V600E

Based on the discovery of the *BRAF*-V600E mutation in virtually all patients with classical HCL ^10^, as well as the success of BRAF inhibitor treatment in melanoma ^45^, it was intuitive that BRAF inhibition is a promising treatment strategy for patients with HCL. The first patient exposed to the BRAF inhibitor vemurafenib indeed showed an immediate and striking response, proving oncogene-dependence and clinical activity ^18, 46^. The dynamics of the response were notable, with a spleen size reduction of more than 6 cm in only 6 days and improvement of blood count (hemoglobin, platelets, and granulocytes) within 1 month ^46^. Soon after the initial report, multiple studies confirmed the efficacy of both vemurafenib ^47– 49^ and (because of availability) later dabrafenib ^50^. One report also presented the use of vemurafenib in a primary refractory patient with severe pulmonary aspergillosis ^51^, where the avoidance of myelotoxicity may be particularly advantageous. Individual dosing regimens of vemurafenib with a minimum of 240 mg twice daily were reported in a series of 21 patients with refractory and relapsed HCL. Both antitumor and side effects were found to be independent of vemurafenib dosing ^52^. Indeed, the initial melanoma study demonstrated response at the 2 × 240 mg level of dosing. Dose finding studies often pick dose levels based on the incidence of side effects rather than target inhibition. Therefore, the data suggest that individual dosing regimens in BRAF-driven cancers warrant reassessment in trials with implications for cost of cancer care.

### Clinical trials

Soon after the discovery of *BRAF*-V600E in HCL, investigators from Italy and the United States designed a single-arm phase II trial testing vemurafenib at standard dosing in relapsed and refractory HCL ^53^. The Italian study (EudraCT 2011-005487-13) comprised 28 *BRAF*-V600E-positive HCL patients of whom 21 experienced early relapse after treatment with purine analogs and 15 were disease refractory to prior therapy. Vemurafenib was given for a median of 16 weeks and was reduced to below 2 × 960 mg in 17 of 28 patients owing to side effects. Drug-related side effects, mostly rash and arthralgia, were generally grade 1–2 and reversible in all patients. Three patients with either cutaneous basal-cell carcinoma (n=2) or superficial melanoma were all successfully treated with a simple excision. Overall response rate was 96%, with rates of 35% CR and 61% PR obtained after a median of 8 and 9 weeks, respectively. Of note, in all patients with CR, minimal residual disease status was evaluated by immunohistochemistry and showed persisting hairy cells in the range of ≤10% at the end of treatment. The median relapse-free survival was 9 months; the relapse-free survival was significantly longer among patients who had a CR than among those who had a PR (19 months versus 6 months). In the same report ^53^, investigators from Memorial Sloan Kettering Cancer Center (MSKCC) reported on 26 patients with HCL who were refractory to purine analogs or who had achieved suboptimal response to purine analogs. Eligible patients received vemurafenib 960 mg twice daily continuously in cycles of 4 weeks for three cycles. Patients with PR or CR with detectable minimal residual disease (MRD) could receive vemurafenib for up to three additional cycles at the discretion of treating physicians. The side effect profile was similar to the Italian study and included rash, photosensitivity, arthralgia, hand-foot syndrome, and febrile neutropenia. Four patients developed new squamous cell carcinoma (n=3) and cutaneous basal-cell carcinoma (n=1), which were successfully resected. Vemurafenib was reduced to 480 mg twice daily in 14 patients. Blood counts recovered in all patients and 41% of the patients achieved CR. At 1 year after response, the cumulative incidence of relapse was 27% (95% CI, 7 to 51) ^53^.

The combined results of these two prospective studies provide good evidence that vemurafenib has potent antitumor activity in patients with relapsed and refractory *BRAF* mutant HCL and confirm the central role of MAP kinase signaling in the pathogenesis of HCL.

### Retreatment with vemurafenib

Despite striking anti-HCL activity of vemurafenib, relapses after drug discontinuation are common, even in patients with CR. Although it cannot be excluded that the selection of highly refractory patients in these studies contributed to the high proportion of early relapses, responses achieved with vemurafenib appear to be less durable than responses achieved with purine analogs.

Relapsing patients can successfully be retreated with vemurafenib ^52, 53^. Furthermore, Bailleux *et al*. reported that vemurafenib at a dose of 240 mg once daily was sufficient to maintain a complete hematological remission after an initial induction treatment with low-dose vemurafenib (2 × 240 mg) ^54^. Whether continuous or intermittent treatment with vemurafenib is superior remains to be shown, but studies in *BRAF* mutant melanoma mouse models suggest that intermittent treatment might be advantageous ^55^. Some genetic lesions have been reported to cause resistance to BRAF inhibition in HCL, including *RAS* ^53^, *PI3K* ^52^, and *IRS1* mutations or loss of *NF1* and *NF2* ^12^. However, resistance formation in HCL seems to be a rare event compared to melanoma patients in whom almost all tumors inevitably develop resistance to vemurafenib within months.

## Perspective

There is compelling evidence that BRAF inhibitors are clinically highly efficacious in HCL. However, the high percentage of incomplete responses and the lack of sustained remissions off drug call for the development of combination approaches. BRAF inhibitors could, for instance, be combined with anti-CD20 monoclonal antibodies to potentially eradicate BRAF inhibitor-resistant hairy cell leukemia cells. Patients with metastatic melanoma have been successfully treated with combined BRAF and MEK blockade ^56^, and the discovery of the reactivation of the MAPK pathway as a probable mode of resistance to vemurafenib in some patients also validates the use of this therapy. Studies for both approaches are on their way.
